# Africa’s emerging role in precision oncology: translational insights from the harnessing functional genomics in cancer research conference, Windhoek, 23-26 September 2025

**DOI:** 10.1186/s12919-026-00387-z

**Published:** 2026-07-28

**Authors:** Charlize L. Rix, Eliud Buchard Buberwa, Justice Amofa, Uvatera Maurihungirire, Rurde Hosea, Linda Ratjama, Graca K. Kandanda, Loide N. Nangolo, Colin Stanley, Georgia Schäfer, Stefano Cacciatore, Richard Banda, Anna Schuh, Vanessa M. Hayes, Devanand Sarkar, Rolf Hansen, David Wedge, Harris Onywera, Nixon Niyonzima, Paulus Mungeyi, Juliet Nabyonga, Laimi Ashipala, Peter Nyarango, John Tabiri Abebrese, Laina Iyambo, Kaenda Rukira, Jean Hategekimana, George M. Jemu, Mariëtte Rothman, Nambumba Amundaba, Helena Musau, Mutsa Takundwa, Jerolen Naidoo, Ireshyn Govender, Tracey Hurrell, Xavier Berthet, Kgomotso Makhaola, Cara Mia Dunaiski, Lameck M. Amugongo, Onesmus Shuungula, Habauka M. Kwaambwa, Percy Chimwamurombe, Taime Sylvester, Precious Simushi, Martin Boys, Alisen Ayitewala, Christian Happi, Lamech M. Mwapagha

**Affiliations:** 1 Department of Biology, Chemistry and Physics, Faculty of Health, Natural Resources and Applied Sciences, Namibia University of Science and Technology, Private Bag 13388, 13 Jackson Kaujeua Street, Windhoek, Namibia; 2 Department of Haematology and Blood Transfusion, Muhimbili University of Health and Allied Sciences, Dar-Es-Salaam, Tanzania; 3 Genomics and Bioinformatics Core Facility Unit, Noguchi Memorial Institute for Medical Research, University of Ghana, Accra, Ghana; 4 Ministry of Health and Social Services, Windhoek Central Hospital, Windhoek, Namibia; 5 Research, Innovation and Partnerships, Namibia University of Science & Technology, Windhoek, Namibia, Private Bag 13388, 13 Jackson Kaujeua Street, Windhoek, Namibia; 6 International Centre for Genetic Engineering and Biotechnology (ICGEB) Wernher and Beit Building (South) Anzio Road, Observatory, Cape Town, 7925 South Africa; 7 Division of Medical Biochemistry and Structural Biology, Department of Integrative Biomedical Sciences, Faculty of Health Sciences, University of Cape Town, Private Bag X3, Observatory, Cape Town, 7935 South Africa; 8 Institute of Infectious Disease and Molecular Medicine (IDM), University of Cape Town, Room 2.1, Wernher & Beit North Building, Anzio Road, Observatory, Cape Town, 7925 South Africa; 9 World Health Organization (WHO), UN House, 38-44 Stein Street, Klein Windhoek, P.O. Box 24449, Windhoek, Namibia; 10 Department of Oncology, University of Oxford, Oxford, UK; 11 Ancestry and Health Genomics Laboratory, Charles Perkins Centre, School of Medical Sciences, Faculty of Medicine and Health, University of Sydney, Camperdown, NSW 2050 Australia; 12 School of Health Systems and Public Health, University of Pretoria, Pretoria, South Africa; 13 Norwich Medical School, University of East Anglia, Norwich, UK; 14 School of Medicine Human and Molecular Genetics, Virginia Commonwealth University (VCU), Richmond, USA; 15 Cancer Association of Namibia (CAN), 90 John Meinert Street, Windhoek West, P.O. Box 502, Windhoek, Namibia; 16 Division of Cancer Sciences, Faculty of Biology, Medicine and Health, The University of Manchester, Oxford Road, Manchester, M13 9PL UK; 17 Manchester Cancer Research Centre (MCRC), The Christie NHS Foundation Trust, Wilmslow Road, Manchester, M20 4GJ UK; 18 Africa Centres for Disease Control and Prevention (Africa CDC), Addis Ababa, Ethiopia; 19 Division of Medical Microbiology, Department of Pathology, Faculty of Health Sciences, University of Cape Town, Cape Town, South Africa; 20 Directorate of Research and Training, Uganda Cancer Institute (UCI), P.O. Box 3935, Upper Mulago Hill Road, Kampala, Uganda; 21 Biotechnology, National Commission on Research, Science and Technology (NCRST), Corner of Louis Raymond & Grant Webster Street, Olympia, Private Bag 13253, Windhoek, Namibia; 22 Faculty of Health Sciences, International University of Management (IUM), Private Bag 14035, 21-31 Hercules Street, Windhoek, Namibia; 23 Namibian Oncology Centre, Corner of Hercules & Pavlov Street, Klein Windhoek, P.O. Box 90518, Windhoek, Namibia; 24 Namibia Institute of Pathology (NIP), Windhoek Central Reference Laboratory, P.O. Box 277, Windhoek, Namibia; 25 AIC Kijabe Hospital, Kijabe Road, Kijabe, Kenya; 26 Synthetic Nanobiotechnology and Biomachines, Synthetic Biology and Precision Medicine Centre, Future Production Chemicals Cluster, Council for Scientific and Industrial Research, Pretoria, South Africa; 27 Bioengineering and Integrated Genomics Group, Future Production Chemicals Cluster, Council for Scientific and Industrial Research, Pretoria, 0001 South Africa; 28 Education, and Innovation, Biomedical Research, Institut Pasteur de Dakar (IPD), 36 Avenue Pasteur, B.P. 220, Dakar, Senegal; 29 African Society for Laboratory Medicine (ASLM), P.O. Box 6059, Addis Ababa, Ethiopia; 30 Biostatistics and Data Sciences Department, Boehringer Ingelheim Pharma GmbH & Co. KG, Biberach an Der Riß, Germany; 31 Department of Software Engineering, Namibia University of Science & Technology, Windhoek, Namibia; 32 Faculty of Health, Natural Resources and Applied Sciences, Namibia University of Science and Technology, Private Bag 13388, 13 Jackson Kaujeua Street, Windhoek, Namibia; 33 School of Natural Resources and Applied Sciences, Namibia University of Science and Technology, Private Bag 13388, 13 Jackson Kaujeua Street, Windhoek, Namibia; 34 Department of Biomedical Sciences, Faculty of Health, Natural Resources and Applied Sciences, Namibia University of Science and Technology, Private Bag 13388, 13 Jackson Kaujeua Street, Windhoek, Namibia; 35 Zambia National Public Health Institute (ZNPHI), P.O. Box 30205, Plot 13 Reedbuck Road, Kabulonga, Lusaka, Zambia; 36 Department of National Health Laboratory and Diagnostic Services, Central Public Health Laboratories (NHLDS-CPHL), Ministry of Health, P.O. Box 7272, Kampala, Uganda; 37 Institute of Genomics and Global Health (Formerly ACEGID), Redeemer’s University, Ede, Nigeria; 38 Department of Biological Sciences, Faculty of Natural Sciences, Redeemer’s University, Ede, Nigeria

**Keywords:** Cancer genomics, Precision oncology, African genomic diversity, Functional genomics, Data governance, Translational medicine

## Abstract

**Purpose:**

Africa is entering a new era of cancer research, driven by renewed commitments to genomic innovation, data equity, and strengthened cancer registry systems. The “*Harnessing Functional Genomics in Cancer Research: Opportunities for Diagnosis and Treatment*” conference, held in Windhoek, Namibia, from 23 to 26 September 2025, convened leading experts to evaluate current progress and identify priorities for advancing cancer genomics and precision oncology across the continent.

**Design:**

This conference synthesis report and narrative review drew on four days of expert-led presentations, interactive panel discussions, and structured delegate engagement sessions. Conference proceedings were documented through session minutes, presenter slide decks, recorded discussions, and post-conference feedback. Perspectives from more than 160 participants representing 21 countries were synthesized into priority thematic areas through an iterative drafting and multi-author review process. Conference findings were integrated with supporting peer-reviewed and grey literature to identify opportunities, challenges, and strategic priorities for advancing precision oncology in Africa.

**Results:**

Discussions highlighted Africa’s unparalleled human genomic diversity and its continued underrepresentation in global genomic datasets, emphasizing the need for African-led genomic discovery and precision oncology strategies. Key priorities included strengthening cancer registries and surveillance systems, expanding genomic infrastructure and workforce capacity, establishing ethical and sovereign data-governance frameworks, and improving the translation of genomic discoveries into clinical practice. Next-generation sequencing, multi-omics approaches, artificial intelligence, and collaborative research networks were identified as important enablers of equitable and sustainable precision oncology implementation across Africa.

**Conclusion:**

Africa’s genetic diversity represents a critical resource for advancing precision oncology. Conference insights, supported by the literature, underscore the need for coordinated investments in genomic infrastructure, cancer surveillance systems, workforce development, ethical data governance, and translational implementation. Advancing these priorities will strengthen genomics-informed cancer prevention, diagnosis, and treatment while positioning Africa as an increasingly important contributor to global oncology innovation.

## Introduction

Cancer is a pathological process in which normal cells escape regulatory control, compromising cellular homeostasis and driving malignant transformation. It represents a complex biological ecosystem shaped by the interplay of germline and somatic mutations, environmental exposures, and aberrant cellular and extracellular signalling [1]. Despite decades of progress, cancer remains one of humanity’s greatest scientific and medical challenges, responsible for nearly one in six deaths worldwide [2]. The era of “one-size-fits-all” oncology is fading, giving rise to precision medicine, an approach that tailors diagnosis and treatment to the unique genetic and molecular profile of each patient. Central to this revolution is functional genomics, the science of interpreting how genes and their interactions drive cancer initiation, progression, and response to therapy [3].

Emerging technologies such as artificial intelligence (AI), machine learning (ML), and advanced bioinformatics are revolutionizing the analysis of large-scale genomic datasets [4]. These tools enable the rapid identification of cancer drivers and molecular signatures, prediction of treatment responses, and detection of therapeutic resistance mechanisms [5]. However, for these innovations to meaningfully improve global cancer outcomes, they must be translated into equitable, real-world applications across all populations, including those historically underrepresented in genomic research. Africa, with its extraordinary genetic diversity and rising cancer burden, stands at a pivotal crossroads. The majority of genomic data driving cancer innovations is derived from non-African populations, limiting the clinical applicability and translational relevance of these findings within African contexts [6]. The distinct biology and clinical presentations observed in African patients highlight the urgent need for African-specific discovery at every level, from genetics to treatment strategies.

While molecular and genomic advances are transforming our understanding of cancer biology, scientific innovation alone is insufficient. Sustainable progress in Africa will depend on policy integration, affordable and regionally applicable diagnostics, new business and access models, robust infrastructure, and health-system strengthening to ensure no patient is left behind. By harnessing and managing functional genomics effectively, Africa can transition from being a consumer of global scientific knowledge to an innovator and a manufacturer of precision oncology solutions with direct and lasting impact. African researchers and clinicians are not merely participants in global oncology but emerging leaders shaping the next chapter of translational cancer research. This article presents key insights and outlines a conceptual roadmap for how Africa can lead a new era of equitable, data-driven precision oncology.

## Conference overview

The “*Harnessing Functional Genomics in Cancer Research: Opportunities for Diagnosis and Treatment*” conference was hosted by the Namibia University of Science and Technology (NUST) from 23 to 26 September 2025 at the Hilton Hotel in Windhoek, Namibia. The conference offered a virtual participation option and was conducted with expert-led presentations and interactive expert panel discussions. The event was supported by the International Centre for Genetic Engineering and Biotechnology (ICGEB), endorsed by the American Society of Clinical Oncology (ASCO), and the African Society for Laboratory Medicine (ASLM). This inaugural conference served as a landmark platform to advance the understanding of functional genomics in cancer research and to explore strategies for translating genomic discoveries into clinical impact.

**Fig. 1:**
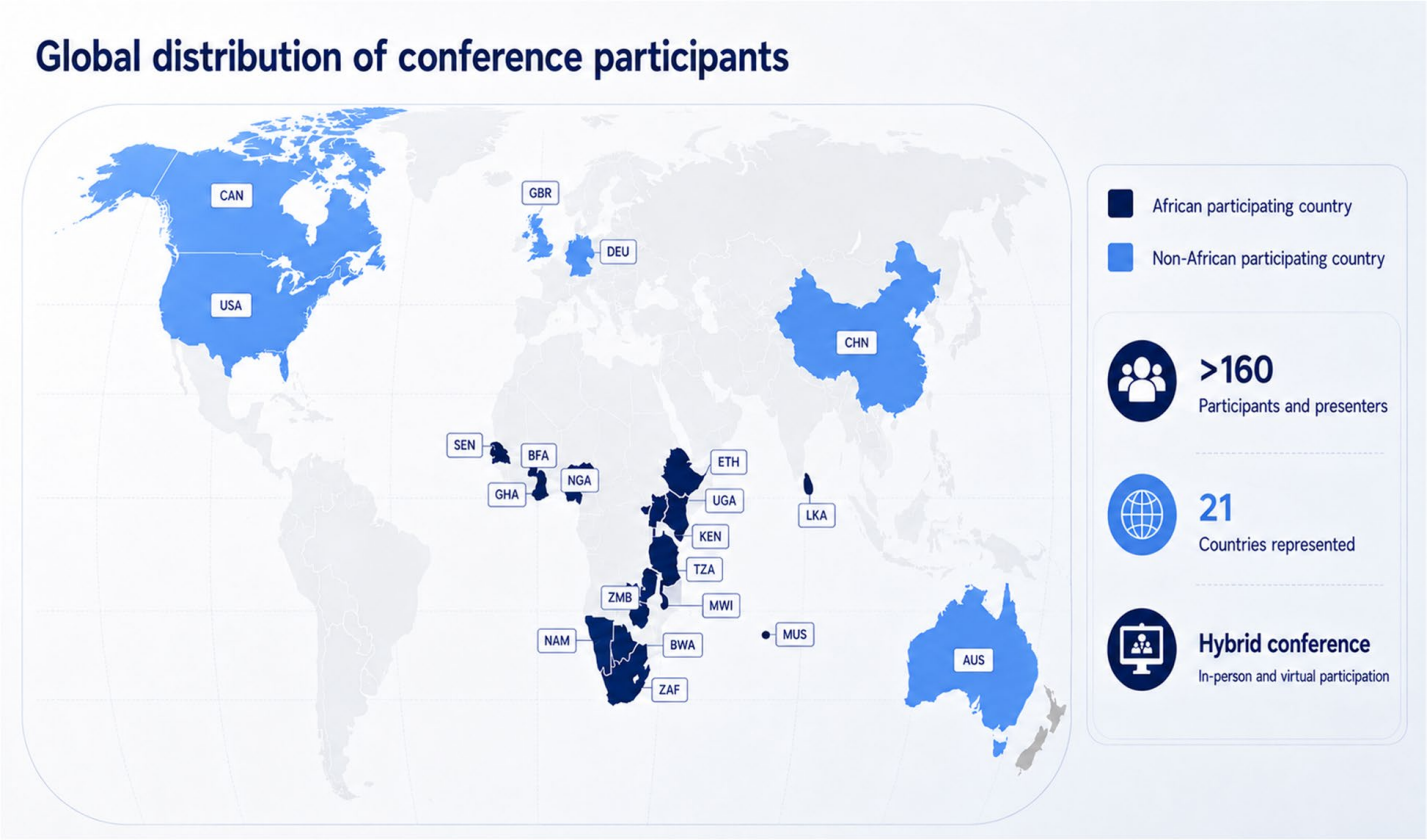
Global distribution of participants in the inaugural “Harnessing Functional Genomics in Cancer Research: Opportunities for Diagnosis and Treatment” conference, hosted by the Namibia University of Science and Technology (NUST), Windhoek, Namibia, 23–26 September 2025. The map illustrates the geographic distribution of participating nations, highlighting the conference’s broad international reach and strong pan-African engagement. Participating countries included Australia, Botswana, Burkina Faso, Canada, China, Ethiopia, Germany, Ghana, Kenya, Malawi, Mauritius, Namibia, Nigeria, Senegal, South Africa, Sri Lanka, Tanzania, Uganda, the United Kingdom, the United States of America, and Zambia. The conference served as a landmark platform for advancing functional genomics and precision oncology research in Africa and beyond

The scale and international reach of the conference were remarkable, attracting over 160 participants and presenters from over 21 countries (Fig. 1), including Australia, Botswana, Burkina Faso, Canada, China, Ethiopia, Germany, Ghana, Kenya, Malawi, Mauritius, Namibia, Nigeria, Senegal, South Africa, Sri Lanka, Tanzania, Uganda, United Kingdom, USA, and Zambia. This diverse representation underscored the conference’s role as a pan-African and global forum for advancing precision oncology. The conference programme comprised 14 thematic sessions spanning cancer epidemiology and genomic diversity, next-generation sequencing and multi-omics, AI and digital pathology, translational medicine, ethical data governance, and strategic investment and capacity development.

## Manuscript approach and methodology

This article is a conference synthesis report and narrative review. It does not describe a primary research study and therefore was not pre-registered. The manuscript integrates thematic content derived from the inaugural “H*arnessing Functional Genomics in Cancer Research: Opportunities for Diagnosis and Treatment*” conference with a targeted supporting literature review.

*Conference Content Capture and Synthesis*. The four-day conference programme encompassed expert-led plenary and parallel sessions, interactive panel discussions, and poster presentations. Conference content was captured through: (i) session minutes compiled by the organizing secretariat; (ii) presenter slide decks submitted to the conference platform; (iii) recordings of plenary and panel sessions; and (iv) a post-conference written feedback process completed by a subset of speakers and session chairs. All 14 thematic sessions were assigned a designated note-taker from the writing committee, and session summaries were circulated to speakers for factual verification prior to manuscript drafting.

*Participant Perspectives and Thematic Consolidation.* All registered participants were eligible to contribute across conference sessions. No formal exclusion criteria were applied, and this report reflects the collective scientific discourse of invited speakers and delegates, including researchers, clinicians, bioinformaticians, public health officials, policymakers, industry partners, and patient advocates. Perspectives expressed during panel discussions, audience question-and-answer sessions, and post-session feedback were captured in session notes and incorporated into thematic summaries. Conference materials were independently reviewed by members of the writing committee, who synthesized content into thematic domains through an iterative drafting process involving three rounds of manuscript review. Themes were identified inductively from conference discussions and mapped to the conference programme structure. Emerging themes were refined through discussion and consensus among the writing committee, with differing interpretations resolved through reference to conference records. Delegate feedback from panel discussions, audience engagement, and post-conference communications informed the refinement and prioritization of themes. As this was a conference synthesis report rather than a formal qualitative study, thematic development did not involve systematic coding procedures, inter-coder reliability assessment, or formal thematic saturation analysis.

*Literature Search Strategy.* A targeted narrative literature search was conducted between October 2025 and April 2026 to supplement and contextualise conference themes. Databases searched included PubMed/MEDLINE, Google Scholar, and African Journals Online (AJOL). Search terms used individually and in combination included: “cancer genomics Africa,” “precision oncology sub-Saharan Africa,” “African genomic diversity,” “population-based cancer registries Africa,” “artificial intelligence cancer diagnostics Africa,” “next-generation sequencing oncology resource-limited settings,” “multi-omics cancer,” “data governance genomics Africa,” “cancer workforce capacity building Africa,” and “liquid biopsy Africa.” Searches were not restricted by language but were limited to publications from 2015 onwards, except for landmark foundational studies (e.g., the first African genome sequences, GLOBOCAN datasets) where earlier publications were included. Grey literature, including WHO reports, World Bank reports, institutional reports, and organisational websites, was reviewed alongside peer-reviewed literature where documentation of ongoing programmes, policy frameworks, or institutional initiatives was required. Inclusion criteria prioritised: peer-reviewed primary research and systematic reviews involving African populations or directly relevant to precision oncology in resource-limited settings; evidence underpinning major recommendations; and current global benchmarks for comparison. Studies were excluded if they lacked methodological transparency, were not available in full text, or were superseded by more recent evidence. Reference lists of key papers were additionally hand-searched to identify relevant citations not captured in the database searches.

## Tracing ancestry to modern trends

### Epidemiology of cancers

Sub-Saharan Africa (SSA), home to over 1.4 billion people, faces a dual burden of persistent infectious diseases alongside a rapidly rising incidence of non-communicable diseases, particularly cancer [7]. This shift is driven by population aging, lifestyle changes, environmental exposures, genetic susceptibility, epigenetic modifications, disruptions in the microbiome and high prevalence of oncogenic infections such as the human papillomavirus (HPV) and Epstein–Barr virus (EBV) [8]. Despite the rising cancer burden, cancer control remains a low priority in many African health systems, mainly due to limited funding, competing priorities such as infectious diseases, and a weak healthcare infrastructure, largely dependent on primary care facilities, with one of the lowest doctor-to-patient ratios globally [7].

In 2022, Africa accounted for $\sim$ 1.2 million new cancer cases and $\sim$ 800,000 deaths, with projections indicating a rapidly escalating cancer burden in the region. While frequently cited figures suggesting SSA will account for over 75% of global cancer deaths should be interpreted contextually (reflecting the convergence of rising incidence, late-stage diagnosis, and health system constraints), the directional trend toward substantially increasing cancer mortality in SSA is well-supported by GLOBOCAN estimates and the Lancet Oncology Commission on Cancer in SSA [9]. The cancer profile across SSA is distinct and highly gendered. Most common malignancies are breast, cervical, prostate, liver, and colorectal cancers, with late-stage diagnosis (Stages III–IV) prevalent in 70%–80% of cases [10]. Consequently, five-year survival rates in Africa frequently fall below 50%, compared with over 80% in high-income countries. Africa faces a critical moment in its cancer control journey, with projected cases of 2.1 million and 1.4 million deaths by 2040 [7].

To alter this trajectory, the continent must integrate genomics, precision health and digital innovation to expand population-based cancer registries and prioritize affordable diagnostics and therapeutics. Progress is already visible through the African Cancer Registry Network (AFCRN), Africa Centres for Disease Control (Africa CDC), African Cancer Coalition, AORTIC and WHO-led initiatives such as the Global Breast Cancer Initiative and the Global Initiative for Childhood Cancer, which support guideline harmonisation, workforce training, and regional centres of excellence [11]. By combining innovation with equitable policy and community engagement, Africa can transition from a reactive and delayed to a proactive, precision-driven model of cancer care.

### African ancestry

The story of human genomics is intrinsically linked to our ancestral origins in Africa. Africa, as the cradle of humanity, harbours the greatest human genetic diversity, containing most allelic variation despite representing only ~19% of the global population; this variation remains largely unrecognized in most commercially available cancer panels to date [12, 13]. The continent’s genomic mosaic mirrors migration, admixture, climate transitions, pathogen exposures, and cultural shifts, directly influencing disease susceptibility, resistance patterns, metabolic pathways, and treatment responses [14]. As populations migrated out of Africa, only subsets of this genetic richness were carried globally, leaving a largely unexplored reservoir of diversity and untapped potential within Africa [14].

Despite Africa’s deep ancestral roots, the genomic era has been characterized by a striking lack of inclusion. The value of African genomic diversity became evident when the first two African genomes, belonging to Archbishop Desmond Tutu (South Africa) and!Gubi (Namibia), were sequenced [15]. From these two individuals alone, researchers identified 1.3 million previously unknown genetic variants, the largest single addition to global databases at the time, revealing just how much of humanity’s genetic story had been missing. Despite ongoing efforts, individuals of African ancestry still account for less than 3% of participants in genome-wide association studies (GWAS) and only 2% of all human genome sequences available globally [16].

The systematic underrepresentation of African genomes remains a major constraint on the accuracy, equity, and clinical translation of genomics-driven medicine. Genomic reference panels, variance databases, analytical algorithms, and diagnostic benchmarks have been derived predominantly from European ancestry datasets, resulting in frequent variant misclassification leading to lowered predictive sensitivity for African genomic variants and limited access to precision therapies [17]. In prostate cancer, for instance, germline testing panels derived from European populations reduce predictive accuracy by more than half for southern African patients [18], yet almost double the frequency for variants of unknown significance (VUS), with whole genome interrogations revealing unknown population-specific gene candidates [19] and pathogenic variants complexity, as well as common risk alleles and polygenic risk scores [20]. In turn, prostate tumour genomes from southern African men exhibit not only a greater tumour mutational burden (TMB), including a higher level of structural variant complexity, but revealed population-specific and clinically relevant molecular taxonomy, driver genes and therapeutic targets. Consequently, the limited applicability of germline testing panels to polygenic risk scores, the gap between cancer drivers and therapeutic targets, and the mismatch of computational tools with variant databases in African populations obscure clinically relevant genes and misinterpret benign, population-specific variants.

Conference delegates underscored how these genomic biases have direct clinical consequences, which is further perpetuated by clinical and biological frameworks that again are not tailored to the African patient. Extending upon the prostate cancer theme, while African men have the highest mortality rates, with southern Africa ranking highest globally [21], patients continue to be diagnosed and risk-stratified using thresholds, such as prostate-specific antigen (PSA) levels, largely calibrated to European cohorts [22]. The application of these benchmarks in African populations increases the risk of misclassification, delayed diagnosis, and suboptimal clinical decision-making [23].

Collectively, these limitations necessitate a transition from a narrow precision-medicine paradigm toward precision health as an integrative framework that situates genomics within broader biological, environmental, and social contexts. African-focused genomic studies are already transforming the field, revealing millions of previously uncharacterized African-specific variants, elevated mutational burdens and tumour heterogeneity, distinct epigenetic architectures [24], and unique environmental signatures that collectively redefine cancer risk, progression, and the future of globally inclusive precision health [25].

## Genomics at the core of precision oncology

Precision oncology is transforming the field of medicine by enabling interventions tailored to the molecular profile of each patient and allowing clinicians to select targeted therapies, thereby increasing efficacy while simultaneously minimizing patient side effects. This approach is seen to move towards a more sophisticated healthcare system aligned with global standards where it reduces the costly and ineffective "trial and error" nature of conventional treatments, which is particularly crucial in resource-limited settings.

When combined with bioinformatics and AI-driven analytics, genomics forms the foundation of precision oncology. AI models integrating genomic data can improve risk stratification, guide targeted screening, and generate predictive frameworks that combine clinical, imaging, and environmental information to support evidence-based treatment decisions [26]. The clinical impact of genomics is illustrated by patient cases:

- In early-stage, luminal (ER +/HER2-) breast cancer, Oncotype DX 21-gene testing has enabled many patients, including older women, to avoid chemotherapy in favour of surgery, radiotherapy, and hormonal therapy [27].
- In stage IV non-small-cell lung cancer, patients with EGFR exon 19 deletions may initially receive erlotinib, but upon progression, repeat sequencing can reveal the T790M resistance mutation, prompting a switch to osimertinib, which often restores disease control [28].

These cases exemplify how genomics shifts oncology from a one-size-fits-all model to truly individualized, evidence-based care, enabling clinicians to identify patients most likely to benefit from specific therapies and to optimize outcomes accordingly. As precision oncology advances, genomics serves not only as its foundation but also as the catalyst driving innovation across the cancer continuum.

## Genomics-driven cancer innovation

The genomic revolution has extended beyond simple gene sequencing to include complex system-level approaches. These advances have given rise to a new era of genomics-driven cancer innovations, illustrating that precision medicine is not a static concept but a continuously evolving paradigm.

### Next-generation sequencing

Next-generation sequencing (NGS) is the technological backbone of all contemporary genomics-driven cancer innovations. NGS enables cost-effective and highly accurate genomic profiling, simultaneously detecting single-nucleotide variants, insertions and deletions, structural and copy-number variations, gene fusions, mutational burden, and microsatellite instability, and ultimately providing a complete view of the tumour mutational burden, mutational signatures and taxonomy beyond single-gene tests [29]. Thus, NGS provides unprecedented insight into the molecular intricacies driving cancer and allows researchers to map the full genetic blueprint of a tumour, against patient-matched inherited genetic profile.

Real-world applications include FoundationOne CDx for whole exome and targeted gene panel sequencing, Guardant360 and Grail Galleri for liquid biopsies supporting early detection and therapy selection, and MSK-IMPACT, which employs a 468-gene panel to match patients with clinical trials [30]. Large-scale efforts such as The Cancer Genome Atlas Program (TCGA) [31] and the International Cancer Genome Consortium Accelerating Research in Genomic Oncology (ICGC ARGO) [32] provide comprehensive genomic and transcriptomic data that have driven numerous discoveries in precision oncology.

Advances in next-generation sequencing (NGS) and molecular profiling have revealed profound genetic differences between tumours of the same histological type and even within individual tumours, highlighting the importance of understanding intra-tumour heterogeneity to design targeted therapies that improve survival and reduce toxicity [33]. Delegates emphasized how NGS has redefined cancer classification by revealing molecular distinctions in tumours that were previously grouped together histologically, enabling new subtypes in cancers like glioma, breast, prostate and lung [34]. It also accelerates research and drug development by identifying novel biomarkers, resistance mechanisms, and therapeutic targets, often linking genotype to phenotype through functional genomics screens.

That said, NGS must be interpreted alongside rigorous histopathological evaluation to distinguish driver from passenger mutations, particularly in African settings characterised by high tumour heterogeneity and late-stage presentation (70%–80% stage III–IV). Morphological assessment provides essential biological context for genomic findings and can refine NGS interpretation, reducing misclassification of VUS. This is underscored by African prostate cancer genomics studies demonstrating a 1.6–2.5-fold higher burden of structural variation compared with other populations [35]. Conference discussions highlighted the importance of pathology-led training initiatives, including hybrid fellowships linked to national cancer registries, exemplified by partnerships between the Cancer Association of Namibia (CAN) and the Namibian Ministry of Health and Social Services (MoHSS), to strengthen multi-omics data validation and accelerate translation into clinical practice [36]. As Africa strengthens its genomics ecosystem, NGS is transitioning from primarily a research tool to a technology with direct clinical relevance [37].

### Integrative multi-omics approaches

Single-omics studies often fail to capture the intricate molecular interplay driving cancer heterogeneity. While historically biased towards genomics, integrative multi-omics incorporates proteomics, metabolomics, and several other layers of biological information to bridge this gap, providing a systems-level perspective essential for accurate diagnosis, prognosis, and treatment selection [38].

As the functional manifestation of the genome, proteomics has become an indispensable tool in precision oncology. By profiling protein expression in accessible biofluids such as plasma or serum, researchers can identify biomarkers for early cancer detection, prognostic stratification, and real‑time monitoring of therapeutic response, as well as predict treatment efficacy and adverse events in populations with unique genetic and metabolic backgrounds, including African cohorts. Given that most existing biomarker panels are derived from non‑African populations, proteomic signatures offer a pathway to regionally tailored, safer, and more effective cancer therapies. Studies from southern Africa illustrate this potential, including a Sequential Window Acquisition of All Theoretical Fragment Ion Spectra Mass Spectrometry (SWATH-MS) proteomic analysis of gallbladder cancer patients of African ancestry that identified dysregulated proteins and plasma-detectable biomarkers linked to extracellular matrix and metabolic pathways [39].

Metabolomics, as the ‘omics’ layer most proximal to phenotype, provides a critical lens through which tumour physiology can be interrogated. In prostate cancer, metabolic profiling has been used to identify hyper-aggressive subgroups defined by specific lipoprotein signatures, which correspond to distinct proteomic and cytokine patterns [40]. Such profiles can highlight inflammatory states that predict response, or resistance, to immunotherapy, depending on immune cell infiltration. Similar metabolomic stratification has been observed in pancreatic cancer, where metabolite markers linked to aggressiveness mirror proteomic and cytokine alterations [41].

According to programme descriptions released by the Council for Scientific and Industrial Research (CSIR), initiatives such as the South African Clinical Multiome Atlas Platform (Clin-MAP), in partnership with The African Cancer Atlas (TACA), seek to generate pan-African integrated multiome datasets and support the development of technologies including 3D patient-derived organoids, single-cell sequencing, and AI-guided target discovery [42, 43]. While these initiatives represent important investments in African cancer research infrastructure, their long-term translational impact remains to be evaluated.

### AI technology and cancer screening

AI technology is emerging as the critical bridge between molecular insights and real-time diagnostic practice, most visibly through the rise of AI-powered pathology and digital pathology systems. AI in pathology refers to computer-based systems that analyse medical images, while digital pathology involves converting physical histology slides into high-resolution digital files for remote viewing and advanced analysis [44]. These technologies are especially critical across many SSA countries, where there are three pathologists per million inhabitants, compared with about 65 per million in developed countries, creating substantial diagnostic pressures [45, 46]. By automating image interpretation, enabling telepathology, and supporting remote specialist consultation, AI-powered digital pathology can help overcome shortages of expertise, long distances, and delays caused by transporting physical slides.

Building on AI’s role in pathology, these technologies are increasingly applied to improve cancer screening across Africa. For example, deep-learning AI algorithms have shown the ability to detect lung nodules on chest radiographs that were initially overlooked by radiologists, suggesting their utility as an added layer of detection in lung cancer screening [47]. Emerging non-invasive approaches, such as AI-assisted iris imaging, have been explored as potential low-cost screening tools; early pilot data from the Ophtascan app in Lubumbashi suggest possible utility for disease screening, including certain cancers, but the evidence remains preliminary and requires independent validation in larger prospective studies before clinical adoption can be recommended [48].

Despite the promise of AI-driven diagnostics, Africa faces major barriers to effective implementation. Most health facilities still rely on paper-based records, making it difficult to generate the high-quality, structured, and interoperable data needed for reliable AI models. Gaps in national registries further hinder epidemiological and genomic research. Infrastructure shortages, such as unstable electricity, limited digital connectivity, and a lack of local expertise in AI, ML, and bioinformatics, remain significant obstacles [49]. The continent’s linguistic diversity, i.e., over 2,000 languages with multiple languages often appearing within a single questionnaire, adds complexity to data collection and AI model development. Trust is another challenge, with low scientific literacy and misinformation affecting community acceptance of new technologies, alongside ethical risks like data exploitation without fair benefit sharing [50].

Africa’s path to sustainable AI adoption relies on building continent-specific datasets and co-developing AI tools tailored to African populations. Africa needs to establish robust data centres, expand public–private partnerships, and conduct prospective trials in high-burden settings. Ultimately, AI should complement, and not replace traditional clinical approaches, supporting a future where precision screening and early detection are accessible and equitable across the continent [51].

## Translational medicine and therapies

The true value of genomic-driven innovations lies in their translation into clinical care. A significant challenge remains in bridging the gap between scientific discovery and routine clinical implementation. Translational medicine and therapies encompass strategies that convert molecular insights into effective diagnostics, targeted treatments, and personalised interventions, i.e., bring precision oncology from the laboratory to the bedside. Delegates noted that while research is being conducted in their hospitals, clinicians often do not receive actionable results in a form that can guide patient care. Strengthening engagement between scientists and clinicians, and ensuring that findings are communicated in clear, clinically relevant terms, is essential to bridge this gap.

Addressing the data deficit and translational gap requires leveraging emerging technologies such as AI, ML, and advanced bioinformatics. AI and ML algorithms enable rapid identification of complex molecular patterns within tumours, predict treatment outcomes, uncover resistance mechanisms, and transform diagnostics by integrating histopathology slides with genomic data or analysing medical images for improved cancer detection [52]. However, to accelerate translation, simplification and integration are key: clinicians need actionable recommendations rather than technical detail. Decision-support tools, standardised reports, and multidisciplinary team meetings facilitate the integration of research findings into clinical practice.

While the ability to read cancer’s genetic blueprint is expanding rapidly, therapeutic options and infrastructure for clinical translation often lag. Delegates proposed a conceptual roadmap to bridge this gap, emphasizing the importance of structured partnerships among researchers, clinicians, and policymakers. Central to this effort is the development of clinical trial units across the continent capable of supporting conventional Phase I–III oncology trials, as well as innovative trial designs and pragmatic studies. Such platforms would enable the simultaneous evaluation of genomic biomarkers, novel therapeutic agents, and repurposed treatments, ultimately accelerating the translation of genomic discoveries into improved patient care.

## Ethical governance and equitable management of genomic data

The rapid expansion of genomics and AI in cancer research has intensified the need for robust frameworks to govern data use, protect patient privacy, and guide sustainable investment. In the context of cancer research, the sensitive nature of genomic and health data demands clear policies on data protection, secure storage, and ethical sharing.

These considerations underscore the urgency of implementing data protection measures to ensure that genomic research benefits local populations ethically and equitably. The misuse or unauthorized disclosure of genomic information risks ethical harm, stigma, or discrimination. Historically, the continent has been subject to “parachute research” and “ethics dumping,” in which external entities exploit local resources without equitable benefits, a concern strongly emphasized by delegates. Ensuring secure, locally managed repositories and trusted research environments (TREs) is therefore both an ethical and strategic imperative, safeguarding patient privacy while enabling equitable scientific collaboration.

Emerging strategies for responsible data governance include federated data models, which allow analyses to be performed locally without transferring raw data across borders, and TREs, which integrate high-performance computing and cybersecurity to facilitate secure collaborative analyses. Tiered access and data segregation are recommended to protect sensitive clinical and genomic information, avoiding direct linkage and enforcing strict security protocols for data entry, analysis, and dissemination. Investments in local storage and computational infrastructure are essential, treating data centres as critical national assets, and enabling researchers to negotiate moratorium periods that allow African investigators early access to datasets prior to global release.

Delegates highlighted the promise of advanced cryptographic approaches for protecting sensitive biomedical data, noting that *“this is the future—homomorphic encryption.”* This technique allows computational analysis to be performed directly on encrypted data without requiring decryption, thereby protecting sensitive genetic information and offering strong privacy guarantees, even when computations are outsourced to untrusted servers [53]. Homomorphic encryption provides a promising framework for privacy‑preserving genomic analysis and cross-institutional collaboration while maintaining strict regulatory compliance.

A key dimension of ethical governance in African genomics research is the recognition of national data ownership and sovereignty, particularly when engaging in large multi-country collaborations. Delegates emphasized that although custodians are responsible for managing and safeguarding datasets, they do not possess unilateral authority over data release, which must remain under the jurisdiction of the originating nation. It was further noted that effective navigation of access and benefit-sharing within large research consortia requires clearly defined, pre-negotiated governance frameworks. These agreements should ensure that local investigators retain substantive decision-making authority regarding data use and dissemination.

Equally critical is the establishment of patient-centred consent processes and public engagement initiatives. Transparent informed consent, culturally sensitive communication, and inclusive public and patient involvement are vital to building trust, particularly in oncology, where complex genomic data are involved. Consent processes should reflect community priorities, consider familial input where culturally appropriate, and ensure participants understand the potential implications of data use [54].

Nevertheless, effective frameworks in cancer genomics must extend beyond regulations and data protection to ensure equitable access to care. Embedding health equity into governance structures ensures that advances in precision oncology benefit all populations, particularly those historically underserved. Delegates stressed the urgent need for equitable diagnostic access in Africa, noting that tests such as Fluorescence in situ hybridization (FISH) and Human epidermal growth factor receptor 2 (HER2) are often unavailable in the public sector and subject to interpretive variability. It was emphasized that patient advocacy can drive governments and pharmaceutical companies to act, arguing that once therapies are accessible, diagnostic services typically follow, ultimately improving cancer care outcomes. A central theme woven throughout the presentations was the necessity of ensuring that precision oncology *"does not remain the privilege of a few but becomes the standard"* of care for all patients.

Ethical data governance and robust policy frameworks are critical for advancing genomics in African cancer research. Establishing secure, locally managed infrastructures and harmonised regulations builds public trust, protects sensitive information, and enables equitable collaboration. At the same time, it is essential to balance data sovereignty with meaningful clinical impact and health innovation: data collection should go beyond publications as an endpoint and actively contribute to improvements in diagnostics, treatments, and patient outcomes. When responsibly managed, clinical data from African cancer research can create a value chain that directly benefits clinicians and patients while guiding strategic investments and capacity-building initiatives, forming the foundation for precision oncology across the continent.

## Shaping and sustaining the future: strategic investments

Delegates highlighted that while rapid advancements in genomics and technology have revolutionized research and healthcare delivery, substantial disparities persist between the Global North and African nations, driven by gaps in workforce capacity, infrastructure, and knowledge systems [55]. Achieving sustainable precision oncology in Africa demands coordinated efforts and approaches that combine human capital development, robust infrastructure, operational stability and cost-effectiveness, as well as collaborative networks.

### Initiatives and collaborative efforts

Delegates discussed several key initiatives shaping genomic research on the continent. This included the Human Heredity and Health in Africa (H3Africa) Consortium, a partnership between NIH, the Wellcome Trust, and the African Academy of Sciences, which builds a continental network of researchers studying the interplay between human genetics, environment, and health [56]. The Genomic Centres of Excellence (GenCoE) initiative, funded by African countries via the World Bank, aims to increase capacity, improve data access, and enhance understanding of non-communicable diseases [57].

Despite the substantial progress made by H3Africa in building research capacity, training African scientists, and establishing biorepositories, significant gaps remain in translating genomic knowledge into clinical tools for oncology [58]. Efforts to develop molecular diagnostics aligned with the WHO Model List of Essential In Vitro Diagnostics, which enable earlier detection of curable cancers, are still limited. Initiatives such as the proposed Three Million African Genomes (3MAG) aim to capture Africa’s extensive genetic diversity and improve representation in global genomic datasets; this reflects broader calls from African researchers to enhance precision medicine on the continent [16].

Additional large-scale initiatives are also addressing cancer research disparities in populations of African ancestry. One such effort is the US Department of Defence (DoD)-funded Health Equity Research Outcomes & Improvement Consortium Prostate Cancer Precision Health Africa1K project [59]. This initiative aims to integrate genomic and clinical data with endocrinological variation (e.g., baseline testosterone differences), exposomic profiles (e.g., exposure to environmental chemicals such as DDT/DDE), and sustained community engagement to generate population-relevant disease models [59]. Similarly, the NIH–Cancer Research UK (CRUK)–funded Grand Challenge project, Social, Ancestry, Molecular and Biological Analysis of Inequalities (SAMBAI), investigates the biological and social determinants underlying disparities in breast, pancreatic, and prostate cancers [60]. However, much of the current dataset and research is focussed on African American and West African populations. Yet these programmes represent substantial investments towards improving precision oncology for populations of African descent.

### Infrastructure and capacity development

Building sustainable precision oncology in Africa depends on both robust technological infrastructure and skilled human capital. Investments in genomic sequencing platforms, high-performance computing, and clinical oncology facilities provide the necessary technological backbone for precision oncology. However, sustainable implementation benefits most from centralised sequencing and high-throughput operations, which maximize resource utilization, reduce redundancy, and maintain cost-effectiveness. Equally important is strengthening expertise in genomics, bioinformatics, and clinical interpretation to ensure innovations are effectively implemented and broadly accessible [61, 62].

Delegates emphasized that shortages of trained pathologists, genetic counsellors, and bioinformaticians remain a key barrier to sustainable precision medicine in Africa. The COVID‑19 pandemic accelerated the launch of virtual genomics training and collaborative laboratory networks that enabled researchers worldwide to connect remotely and helped African students and scientists access high‑performance computational infrastructure and bioinformatics platforms otherwise unavailable in their home countries [63]. These actions guided by accreditation and quality assurance standards from organizations such as ASLM, are critical for long-term success. Additional effort is being made to institutionalize genomics, bioinformatics, and computational biology at African universities and medical schools, supporting centralised sequencing hubs and regional collaborations.

Africa’s greatest resource for advancing cancer genomics is its young people and substantial material resources. Delegates agreed that leveraging both talent and funding, while being deliberate in investments can build sustainable infrastructure and genomic capacity.

### Operational sustainability and cost-effectiveness

Investments in technology and talent must be complemented by efficient resource management, scalable models, and strategies to reduce costs without compromising quality. Prioritizing sustainable operations ensures that genomic services remain accessible, reliable, and equitable, maximizing the benefits of both human and material resources across the continent.

Sequencing costs have decreased dramatically, from over $100 million for the first human genome in 2001 to approximately $500 per genome today [64]. The declining cost of sequencing has transformed precision oncology from a theoretical possibility to a practical reality. Operational sustainability should be heightened through centralised sequencing, high sample throughput, and strategic partnerships that reduce redundancy and maximize utilization of existing infrastructure. Delegates highlighted that focus should be on sequencing germline variants that inform pathogenicity in cancer, rather than the entire genome, which reduces both the financial burden and the data-analysis complexity, making precision oncology more accessible and practical for routine clinical use.

Another cost-effective practice discussed at the conference includes liquid biopsy for paediatric Burkitt lymphoma: micro-costing analyses showed pathology costs of $173 per test compared to $600 for NGS technology when evaluating long-term outcomes and survival, this intervention’s cost-effectiveness fell well within the WHO bracket of three times GDP per capita, highlighting the value of genomics-informed care [65, 66]. However, even when cost-effective genomic tests exist, patients may still be unable to access them due to reimbursement restrictions and upfront payment requirements. Thus, operational sustainability is also shaped by the realities of medical aid coverage. As emphasized during the conference, these financial barriers directly undermine the potential impact of precision oncology in Africa.

Shaping and sustaining the future of cancer genomics in Africa requires not only deliberate investments in infrastructure and workforce but also leveraging existing sequencing infrastructure and regional hubs rather than establishing parallel systems, which can fragment resources and reduce efficiency. Thereby, African nations can “*create a self-sufficient ecosystem where people can collaborate*” and translate genomic discoveries into equitable clinical care and improved population health outcomes. However, realizing this vision will require careful consideration of the practical, financial, and systemic realities that influence implementation across diverse African contexts.

## Critical appraisal and implementation considerations

The ambitions articulated during the conference are compelling, yet their translation into routine practice faces substantial structural, financial, and operational challenges. Implementation feasibility varies considerably across time horizons. In the near term, priorities such as strengthening cancer notification systems, expanding cancer registry infrastructure, establishing data-governance frameworks, and integrating genomics into national cancer control strategies are achievable with targeted investment and political commitment. Longer-term goals, including regional multi-omics platforms, clinically integrated AI-assisted diagnostics, and genomically informed treatment pathways, will require sustained funding, workforce development, infrastructure expansion, and policy alignment. A phased implementation approach supported by established implementation science principles may facilitate realistic benchmarking, adaptive resource allocation, and continuous evaluation of progress. To facilitate translation of conference recommendations into practice, proposed implementation priorities are summarized according to short-, medium-, and long-term implementation horizons (Table 1).

**Table 1:** Proposed implementation priorities for advancing cancer genomics and precision oncology in sub-Saharan Africa. Priorities are organized according to short-, medium-, and long-term implementation horizons, together with illustrative monitoring indicators and key stakeholder groups identified during conference discussions

Time Horizon	Strategic Priority	Example Monitoring Indicators	Key Stakeholders
**Short-term (≤ 2 years)**	Strengthen cancer notification and registry integration within national surveillance systems	Proportion of health facilities reporting to cancer registries; registry completeness and reporting timeliness	Ministries of Health; National Cancer Registries; WHO AFRO
	Establish harmonised data-governance, data-sharing, and benefit-sharing frameworks	Number of countries with approved governance frameworks; number of operational data-sharing agreements	African Union; AUDA-NEPAD; National Ethics Committees; Data Protection Authorities
	Expand access to molecular diagnostics for priority cancers in tertiary referral centres	Proportion of tertiary centres offering molecular testing; median turnaround time from biopsy to molecular diagnosis	National Cancer Programmes; Academic Medical Centres; Pathology and Laboratory Services
**Medium-term (2–5 years)**	Develop regional genomic sequencing and bioinformatics hubs	Number of operational sequencing hubs; annual sample throughput; average turnaround time	Regional Economic Communities (ECOWAS, SADC, EAC); African Union; Research Funders
	Establish federated multi-omics and clinical data platforms	Number of participating institutions; number of interoperable datasets; proportion of samples linked to clinical metadata	Research Consortia (e.g., H3Africa); Ministries of Health; Bioinformatics Centres
	Strengthen genomics workforce development and clinical trial capacity	Number of accredited training programmes; number of genomics specialists trained; number of operational clinical trial units	Universities; Medical Schools; ASLM; National Medical Councils; WHO AFRO
**Long-term (5–10 years)**	Establish sustainable pan-African biobanking infrastructure linked to clinical and genomic data resources	Number of participating biobanks; geographic coverage; proportion of samples with longitudinal follow-up data	Research Institutions; National Governments; International Funding Partners
	Improve representation of African populations in genomic reference resources and clinical interpretation pipelines	Inclusion of African reference datasets in genomic databases and analytical pipelines; number of African-derived variants curated in public repositories	Genomics Research Networks; Database Curators; Industry Partners
	Integrate genomics-informed diagnostics and treatment pathways into national health systems	Number of countries incorporating genomic diagnostics into national policies or essential diagnostics lists; reimbursement coverage for molecular testing	Ministries of Health; Health Insurance Agencies; WHO; Africa CDC

*Abbreviations*: *Africa CDC* Africa Centres for Disease Control and Prevention, *ASLM* African Society for Laboratory Medicine, *AUDA-NEPAD* African Union Development Agency–New Partnership for Africa’s Development, *EAC* East African Community, *ECOWAS* Economic Community of West African States, *H3Africa* Human Heredity and Health in Africa, *SADC* Southern African Development Community, *WHO AFRO* World Health Organization Regional Office for Africa

Cost and infrastructure limitations remain among the most significant barriers to implementation. Although sequencing costs have declined substantially, whole-genome and multi-omics profiling remain expensive relative to the health budgets of many SSA countries. Conference participants highlighted several pragmatic entry points, including targeted molecular testing, selected liquid biopsy applications, and AI-assisted pathology workflows, which may offer more feasible near-term opportunities than comprehensive genomic profiling. Expanding access to these technologies will require sustainable financing models, improved reimbursement mechanisms, and strategic investments in laboratory, computational, and digital infrastructure.

Emerging technologies such as AI and integrative multi-omics approaches offer considerable promise but require careful evaluation before widespread clinical adoption. AI models developed primarily using non-African datasets may underperform in African populations if not locally validated, while inconsistent electricity supply, limited digital connectivity, and shortages of technical expertise may constrain implementation. Similarly, multi-omics approaches remain analytically complex and dependent on high-quality biospecimens, robust laboratory standards, and population-specific reference datasets that are still being developed for many African populations. Consequently, these technologies are currently most appropriate within research and specialist settings, with broader clinical implementation requiring phased capacity development, harmonised standard operating procedures, quality assurance systems, and prospective validation studies.

Equity considerations must remain central to implementation efforts. Increased genomic data generation without corresponding investments in analytical capacity, clinical infrastructure, and governance frameworks risks reinforcing existing disparities rather than reducing them. Likewise, an exclusive focus on advanced technologies could divert resources from foundational interventions, including cancer awareness programmes, earlier diagnosis, pathology services, and access to standard treatments that continue to have substantial population-level impact. Ensuring that genomic innovation complements rather than replaces essential cancer services will be critical for achieving equitable and sustainable precision oncology across SSA.

Taken together, these implementation realities underscore that the future of cancer genomics in Africa will depend not only on scientific advancement, but also on how effectively innovation is integrated into broader health systems, governance structures, and population health priorities. Sustainable progress will require coordinated action across research, clinical practice, policy, infrastructure, and workforce development.

## Conceptual roadmap for precision oncology in Africa

**Fig. 2:**
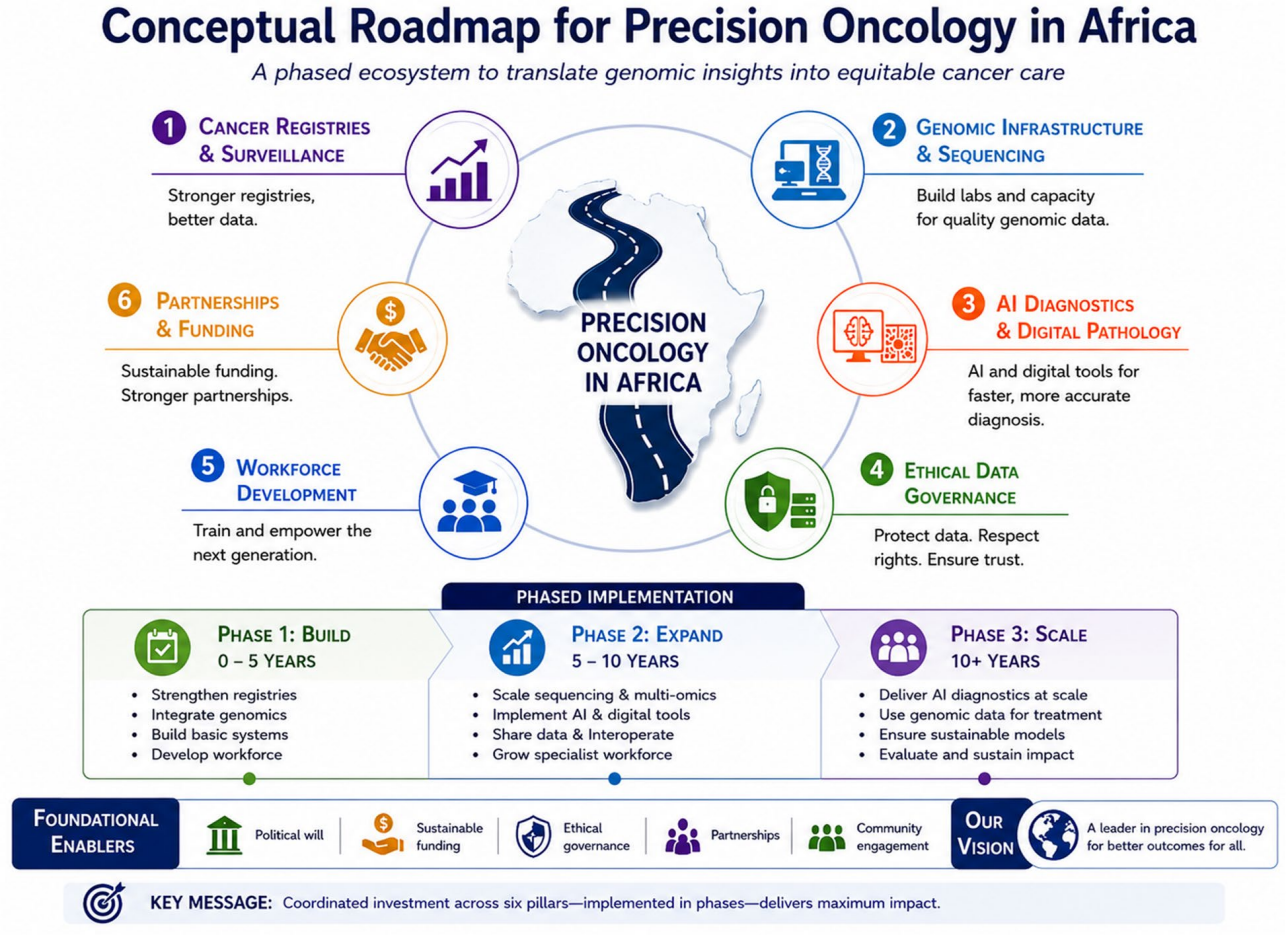
Conceptual roadmap for precision oncology in Africa. The figure illustrates a coordinated framework for advancing precision oncology across Africa, with genomics positioned at its core. Six interconnected pillars—registries and surveillance, genomic infrastructure, AI-enabled diagnostics, ethical data governance, workforce development, and partnerships and funding—define the foundational requirements for translating genomic innovation into equitable cancer care. The roadmap outlines staged implementation from foundational system strengthening and capacity building to long-term scaling of multi-omics, AI-assisted diagnostics, and genomically informed healthcare

The “*Harnessing Functional Genomics in Cancer Research: Opportunities for Diagnosis and Treatment*” conference highlighted Africa’s unique position at the intersection of genomic innovation and public health, offering a conceptual roadmap for the continent’s future in precision oncology. This roadmap positions genomics as the central pathway driving cancer innovation across the African continent. Africa’s rich genetic diversity is not merely a scientific variable but a foundational asset. Understanding this diversity is essential for advancing cancer prevention, diagnosis, and treatment, all of which increasingly rely on genomic insights to enable personalised interventions. However, genomic progress in Africa cannot occur in isolation. There are interconnected pathways representing essential factors that must be integrated to translate genomic discoveries into meaningful health impact (Fig. 2).

Key outcomes from the conference underscore the need for coordinated and multi-disciplinary action. Formalizing cancer notification systems, institutionalizing national cancer registries within government structures, and securing sustainable and multi-source funding emerged as immediate priorities. These foundational steps will enable the collection of high-quality, comprehensive data, which is an essential prerequisite for translational research, evidence-based policy-making, and monitoring population health. Strategic investments in capacity building, from training clinicians and scientists to developing laboratory infrastructure, will ensure that innovation is not confined to a few centres, but benefits entire populations.

The commitments made by delegates at the conference reflect a collective vision to transform cancer genomics into actionable knowledge that directly informs clinical decision-making and improves patient outcomes across African populations. Success can be tracked through concrete metrics, including increased completeness and timeliness of cancer registration, growth in genomics-driven clinical trials, expanded research outputs addressing ancestry-specific risk factors, and demonstrable improvements in patient outcomes at national and regional levels. Collaboration across African nations and with global partners remains a cornerstone for achieving these metrics, fostering knowledge exchange, capacity sharing, and joint advocacy efforts.

For Africa, the next decade, and beyond, will be defined by how effectively insights from the conference are applied, turning health science into equitable healthcare, integrating precision medicine into national strategies, and sustaining momentum through strategic investment, governance, and collaboration. Ultimately, the conference has set a precedent: By harnessing and managing functional genomics effectively, African researchers and clinicians are not merely participants in global oncology but emerging leaders shaping the next chapter of translational cancer research. Africa is no longer considered a recipient of global scientific knowledge and innovation, but rather, a producer of precision oncology solutions with direct and lasting impact.

## Limitations

As described in the methodology, this conference synthesis report did not employ formal qualitative research methodologies such as systematic coding procedures, inter-coder reliability assessment, or thematic saturation analysis. The themes presented reflect discussions and perspectives from conference participants and invited speakers and may not fully represent all stakeholder groups, geographic regions, or health-system contexts across Africa. Furthermore, conference priorities were inherently influenced by the programme structure and expertise of invited speakers. The supporting literature review was narrative rather than systematic and may not have captured all relevant evidence. Several recommendations remain conceptual and were not evaluated through formal implementation, economic, or outcomes-based analyses. Finally, given the rapidly evolving nature of genomics, artificial intelligence, and precision oncology, some technologies, policies, and implementation priorities discussed may evolve as new evidence emerges.

## Data Availability

The materials supporting this conference synthesis include conference session notes, presentation materials, recordings of plenary and panel discussions, and anonymized post-conference feedback. These materials are not publicly available but may be made available from the corresponding author upon reasonable request and subject to applicable conference permissions and data-sharing considerations.
